# Chronic Liver Disease: Latest Research in Pathogenesis, Detection and Treatment

**DOI:** 10.3390/ijms241310633

**Published:** 2023-06-25

**Authors:** Silvia De Siervi, Stefania Cannito, Cristian Turato

**Affiliations:** 1 Department of Molecular Medicine, University of Pavia, 27100 Pavia, Italy; 2 Department of Clinical and Biological Sciences, University of Turin, 10125 Turin, Italy; stefania.cannito@unito.it

Chronic liver disease (CLD) is a major global health threat and has emerged as a leading cause of human death. There are several causes behind the development of CLDs, but some of the most common include alcohol abuse, obesity/metabolic disease, autoimmune hepatitis, and viral hepatitis (HBV and HCV) [1].

Among them, non-alcoholic fatty liver disease (NAFLD) is the primary cause, representing more than 50% of cases. NAFLD is characterized by the intracellular deposition of lipids in hepatocytes, often associated with a wide spectrum of metabolic abnormalities, such as obesity, diabetes, dyslipidemia, hypertension, and insulin resistance [2]. NAFLD represents a different range of liver disease, from a bland fatty infiltration to progressive non-alcoholic steatohepatitis (NASH), a more severe condition that includes inflammation and additional hepatocyte damage, and can progress to cirrhosis. Consequently, these patients are at high risk of developing hepatocellular carcinoma (HCC) [3].

Due to high incidence and mortality, there is a need to better understand the mechanisms underlying the transition process from a healthy liver to end-stage disease in order to predict patients at a high risk of developing HCC, detect HCC at an early stage, and predict the evolution, recurrence, and outcome after treatment.

This Special Issue entitled “Chronic Liver Disease: Latest Research in Pathogenesis, Detection and Treatment” of the *International Journal of Molecular Sciences* includes a total of 11 contributions: 6 original articles and 5 reviews providing new information about the role of inflammation in the pathogenesis of chronic liver disease and describing possible novel targets for diagnosis and treatment.

NAFLD progression is affected by the balance between pro- and anti-inflammatory stimuli. The up-regulation of several pro-inflammatory cytokines characterizes inflammation in NAFLD/NASH development. In this connection, Winarto et al. [4] reported that fucoxanthin, a naturally derived carotenoid compound that simultaneously has anti-obesity and anti-inflammatory effects, can alter PPAR signaling pathways and control inflammation and the immune response by regulating macrophages. In fact, the authors showed that fucoxanthin supplementation successfully suppressed IL-1β, TNF-α, COX-2, and iNOS in a high-fat diet (HFD)-induced obese mouse model, and also suppressed pro-inflammatory factors in the RAW 264.7 macrophage cell line via the NF-κB and MAPK signaling pathways, displaying its promise in the treatment and prevention of NAFLD. Moreover, fucoxanthin can exhibit anti-fibrogenic activity that prevents NASH development [4].

Chronic low-level inflammation with abnormal production of inflammatory adipocytokines in adipose tissue is a hallmark of obesity and contributes to various metabolic disorders at the base of NAFLD onset, such as type 2 diabetes (T2D). Focusing on the deregulation of hepatic glucose production through gluconeogenesis, which leads to hyperglycemia and T2D, Qiaio et al. [5] investigated that the ablation of the adaptor protein Sam68 in hepatocytes can protect mice from high-fat diet-induced hyperglycemia and significantly increase hepatic and peripheral insulin sensitivities, representing a potential therapeutic target for T2D.

Because NASH-caused liver injury is a complex process involving multiple cell types, intercellular communication mediated by extracellular vesicles (EVs) is gathering significant traction. During lipotoxicity, EVs are released in large quantities from different hepatocyte or hepatic non-parenchymal cells, playing a key role in the transmission of information between different cell types in the liver. They can change the function of target cells and activate different pathways, promoting the pathogenesis and development of NAFLD/NASH. In particular, small EV exosomes contain various cellular molecules, including proteins, messenger RNAs (mRNAs), and microRNAs (miRNAs). A summary of the current knowledge regarding the involvement of exosomal miRNAs in NASH progression was reported in the review by Qi and Lai [6]. Based on mouse model experimental studies and limited clinical observations, they revealed that various exosomal miRNAs are associated with liver steatosis, hepatocyte ballooning, inflammation, and fibrosis, becoming potential circulating biomarkers for clinical diagnosis and the treatment of NAFLD/NASH.

MiRNA profiles may also serve as potential HCC molecular markers. The evaluation of several plasma homo sapiens miRNAs as potential biomarkers for HCC in chronic HCV patients with liver cirrhosis, especially AFP-negative HCC cases, was the innovative subject of the study of Eldosoki et al. [7] that demonstrated the link between these miRNAs to insulin metabolism, inflammation, dyslipidemia, and tumorigenesis in the HCC patients’ group, as well as to an up-regulated independent risk factor for the emergence of HCC from cirrhosis in the chronic HCV patients.

Since an up-regulated long non-coding RNA (lncRNA) signature was reported to be associated with increased immune infiltration and worse overall survival in HCC, lncRNAs can also represent putative biomarkers. Kunadirek et al. [8] observed an alteration in lncRNAs expressed by the peripheral blood mononuclear cells (PBMCs) of patients with HBV-related HCC.

In particular, in this study, a transcriptomic analysis highlighted three up-regulated differentially expressed lncRNAs, including MIR4435-2HG, SNHG9, and lnc-LCP2-1, and one down-regulated lncRNA, lnc-POLD3-2, mainly associated with carcinogenesis and immune responses, demonstrating that circulating MIR4435-2HG and lnc-POLD3-2 either alone or in combination could be used as sensitive biomarkers of HCC detection in patients with chronic HBV infection.

Regarding the steatotic situation that characterizes CLDs, including NAFLD/NASH and HCV, a relevant role is played by the pathologic accumulation of micro- or macrovesicular lipid droplets (LDs) in hepatocytes. Concerning this, a study by Schelbert et al. [9] demonstrated the importance of LD-associated proteins of the perilipin family in steatotic liver diseases. Using a large collection of human liver biopsies with HCV of different grades and stages, they showed that perilipins 1 and 2 localized to LDs of hepatocytes, correlating with the degree of steatosis and specific HCV genotypes, but not significantly with the HCV viral load. Moreover, they observed perilipin 1- and 2-positive microvesicularsteatotic foci in 36% of HCV liver biopsies and also in chronic hepatitis B, autoimmune hepatitis, and mildly steatotic or normal livers, but less or none in the normal livers of younger patients.

A special role in the promotion of liver fibrosis during the development of chronic inflammation is given to interleukine-17A (IL-17A), which ensures, with IL-22, the establishment of type 3 inflammation in hepatic tissue and provides a massive influx of other immune cells, causing fibrosis activation. The pathogenic role of IL-17A has been suggested in different etiological causes of liver fibrosis in animal models; however, there is some evidence of its protective role in acute liver conditions. Using human liver slices, Kartasheva-Ebertz et al. [10] investigated the pro-fibrotic and pro-inflammatory role of IL-17A in liver tissue, observing a higher concentration of this target either in the early stage of fibrosis or in the total absence of fibrosis. The authors thus assumed that IL-17A production is not a critical element that defines fibrotic events but is possibly necessary to initiate the pro-fibrotic reaction, becoming a potential precursor marker of liver fibrosis and a target for anti-fibrotic liver treatment.

The immune system also plays a central role in the pathogenesis of Biliary Atresia (BA), a severe obliterative cholangiopathy in early infancy that is by far the most common cause of surgical jaundice and the most common indicator for liver transplantation in children. An illustration of the clinical characteristics of different BA subtypes was the leading argument of the review by He et al. [11], who also highlighted the involvement of inflammation in the development of this disease. In general, overexpression of intercellular cell adhesion molecules on cholangiocytes can promote these cells to produce immunologic costimulatory factors that activate antigen-presenting cells in portal tracts, followed by the infiltration of activated natural killer cells that induce up-regulation of IL-1R1 and NLRP3, facilitating Th1 cell-mediated release of cytokines and chemokines.

The activation of inflammasome and pyroptosis, processes involved in the progression of liver fibrosis and its end-stage cirrhosis, is extensively reviewed by Lucas-Ruiz et al. [12], who discussed the expected role of machine perfusion (MP) technology to modify the injury response related to inflammasome activation. The inhibition of the inflammasome may have a prominent place among the different strategies. The authors reported that pharmacological inhibitors against several steps of the inflammasome activation pathway are currently being tested. Ex vivo MP could provide an excellent platform for therapeutic interventions during ex vivo liver preservation, facilitating the use of specific drugs or even gene silencing with small interference RNA.

In addition, pediatric autoimmune hepatitis (AIH) is characterized by immune-mediated hepatocyte injury destroying liver cells, causing inflammation, liver failure, and fibrosis. After profiling a list of proteins involved in relevant mechanistic pathways for liver diseases and immune processes in AIH pathogenesis, the pilot study performed by Sîrbe et al. [13] identified markers that could potentially lead to new diagnostic and therapeutic tools.

The review of Jastrzębska et al. [14] has deepened some preclinical investigations into the effect of chronic cocaine exposure on hepatic levels of cytochrome P450s, suggesting that the action of these addictive substances may affect its oxidative metabolism and the metabolism of endogenous substrates and other co-administered drugs, leading to hepatotoxicity.

In conclusion, the role that inflammation plays in chronic liver disease and the scarcity of specific treatments justify ongoing efforts to identify more accurate diagnostic techniques and targeted therapies. This Special Issue is intended to provide readers with an update on the most recent perspectives in the study of chronic liver disease.
